# *Rosa Roxburghii* Tratt Fruit Extract Prevents Dss-Induced Ulcerative Colitis in Mice by Modulating the Gut Microbiota and the IL-17 Signaling Pathway

**DOI:** 10.3390/nu15214560

**Published:** 2023-10-27

**Authors:** Xingjie Li, Yihan Ling, Xiaoyi Huang, Ting Zhou, Shouxun Wu, Shuwen Zhang, Heting Zhou, Yuhong Kang, Liqun Wang, Xiaomeng Wang, Wenya Yin

**Affiliations:** 1 West China School of Public Health and West China Fourth Hospital, Sichuan University, Chengdu 610041, China; lixingjie6588@163.com (X.L.); tparks@163.com (Y.L.); tingzhou1002@163.com (T.Z.); wushouxun2021@163.com (S.W.); 15111710429@163.com (S.Z.); zht200722@163.com (H.Z.); kangyuhong0107@163.com (Y.K.); wangliqun8393@163.com (L.W.); m15559300325@163.com (X.W.); 2 Department of Clinical Nutrition, Sichuan Provincial People’s Hospital, University of Electronic Science and Technology of China, Chengdu 610072, China; xiaoyi_huang1999@163.com

**Keywords:** ulcerative colitis, *Rosa roxburghii* Tratt fruit, natural products, extract, inflammation, oxidative stress, gut microbiota, transcriptomics

## Abstract

Ulcerative colitis (UC) is a non-specific inflammatory bowel illness characterized by intestinal mucosal barrier degradation, inflammation, oxidative damage, and gut microbiota imbalances. *Rosa roxburghii* Tratt Fruit extract (RRTE) was extracted from *Rosa roxburghii* Tratt fruit, exhibiting an excellent prevention effect against UC; RRTE could prevent the damage of DSS-induced human normal colonic epithelial (NCM 460) cells, especially in cell viability and morphology, and oxidative damage. Additionally, in UC mice, RRTE could limit the intestinal mucosal barrier by increasing the expression of intestinal tight junction proteins and mucin, reducing inflammation and oxidative damage in colon tissue. More importantly, RRTE can increase the abundance of beneficial bacteria to regulate gut microbiota such as *Ruminococcus*, *Turicibacter*, and *Parabacteroides*, and reduce the abundance of harmful bacteria such as Staphylococcus and Shigella. Furthermore, transcriptomics of colonic mucosal findings point out that the beneficial effect of RRTE on UC could be attributed to the modulation of inflammatory responses such as the IL-17 and TNF signaling pathways. The qPCR results confirm that RRTE did involve the regulation of several genes in the IL-17 signaling pathway. In conclusion, RRTE could prevent DSS-induced damage both in vitro and in vivo.

## 1. Introduction

Ulcerative colitis (UC) is one of the more common subtypes of non-specific intestinal inflammatory diseases, which involves the rectum and colon, manifested as varying degrees of weight loss, abdominal pain, bloody diarrhea and other symptoms [1]. The disease has a protracted course, is prone to relapse and causes cancer, which reduces patients’ quality of life and raises their psychological burden [2]. Epidemiological data show that UC is now a worldwide health problem; in particular, the incidence and prevalence in Asian countries, which were traditionally low, are showing rapid increases. In addition, onset of UC at a younger age is more common, including adolescent and very early onset disorders [3]. In addition, gastrointestinal tract injury, especially UC, was observed in some patients during the COVID-19 pandemic [4]. The pathogenesis of UC is yet to be clarified, and dysregulation of the body’s immunity, alterations in the gut microbiota, impairment of the epithelial barrier, genetics, and the environment may be risk factors for this disease [5]. Although aminosalicylates, corticosteroids, immunosuppressants, and biologics can alleviate disease symptoms, they can cause side effects such as nausea and vomiting, liver and kidney damage, and drug resistance; additionally, the expensive medications add to the patients’ financial burden [3,6]. The treatment of UC remains challenging, and there is an urgent need to find highly effective and safe, yet cost-effective therapeutic modalities to prevent and treat UC.

Natural products derived from herbs, medicinal plants and functional foods have been favored by researchers for their bioactivities such as anti-inflammatory, anti-oxidant, and immunomodulatory properties, combined with high safety and low cost [7,8,9]. Flavonoid extracts of okra flowers have been found to alleviate DSS-induced UC by protecting the intestinal barrier and modulating the NF-κB signaling pathway and intestinal microbial composition [10]; cherry polyphenol extract reduces oxidative damage and pro-inflammatory cytokine release, protects intestinal barrier function, and inhibit the Wnt/β-Catenin signaling pathway to protect from the UC [11]; the proprietary Chinese medicine Zhuling Jianpi capsule may rely on its flavonoids, terpenoids and other compounds to alleviate UC [12]. Therefore, natural products that are rich in polyphenols, flavonoids, and terpenoids may be promising alternative therapeutic agents for UC [13]. 

*Rosa Roxburghii* Tratt (RRT) is a medicinal and food plant which primarily is grown in southwest China. It has a variety of pharmacological activities such as antioxidant, anti-inflammatory, immunomodulatory, anti-tumor, and anti-atherosclerotic activities, which may be due to its rich content of vitamin C (VC), superoxide dismutase (SOD), polyphenols, flavonoids, and terpenoids, and other compounds [14,15]. As early as in the Qing Dynasty, the “*Ben Cao Gang Mu Shi Yi*” was compiled by Xuemin Zhao which recorded that RRT has the ability to promote digestion, treat diarrhea, and have other effects [14]. *Rosa roxburghii* Tratt Fruit Polyphenols may inhibit lung tissue inflammation in mice with acute lung injury by suppressing hyperactivation of complement and coagulation cascades [16]. *Rosa roxburghii* Tratt Fruit Vinegar can alleviate dyslipidemia, liver inflammation and oxidative damage in high-fat diet mice; further, it can regulate the composition of gut microbiota, and plays a role in alleviating obesity and its complications [17]. Polyphenol-rich *Rosa Roxburghii* Tratt extract alleviates DSS-induced human normal colonic epithelial (NCM 460) cells damage but has not been demonstrated in animal models [18]. Based on the compositional characteristics, abundant pharmacological actions, and medicinal history of RRT, we anticipated that it has the potential to alleviate UC, but this still needs comprehensive verification.

RRT fruit is spiny, so it is difficult to consume directly; moreover, the content of active substances obtained through direct consumption is limited. Based on the composition characteristics of RRT and the anti-inflammatory potential of polyphenols, flavonoids, and terpenoids mentioned above, this study aims to extract these components as extraction targets to prepare extracts. We used alcohol extraction combined with alkaline solubilization and acid precipitation to enrich them in RRT fruits, and preliminarily analyzed the possible active components and antioxidant activities of *Rosa roxburghii* Tratt fruit extract (RRTE). We used dextran sulfate sodium (DSS) to establish the NCM 460 cell injury model and the animal model of UC to comprehensively explore the relief effect and underlying mechanism of RRTE in UC. Our research brings up a new avenue for the development of medications for the prevention and treatment of UC, and promotes the development and utilization of the *Rosa Roxburghii* Tratt industry.

## 2. Materials and Methods

### 2.1. Chemicals and Reagents

The variety of RRT used in this study was “Guinong 5”, grown in the *Rosa Roxburghii* Tratt base of Shengjing Street, Panzhou City, Guizhou Province, at 104°24′47″ E, 25°36′50″ N. Cellulase, anhydrous ethanol, and ethyl acetate were purchased from Chron Chemicals (Chengdu, China); rutin, ursolic acid, and chlorogenic acid were purchased from Desite Biotechnology (Chengdu, China); the freeze drier was obtained from Shanghai Zhengqiao Scientific Instrument Co., Ltd. (Shanghai, China); DMEM (Gibco) was purchased from Thermo Fisher Biochemical Product (Beijing, China) Co., Ltd. (Beijing, China); the Cellular Reactive Oxygen Assay Kit was obtained from Beyotime Biotechnology (Shanghai, China); dextran sulfate sodium (Mw 36–50 kDa) was purchased from MP Biomedicals, LCC (Solon, OH, USA); the animal tissue total RNA extraction kit was obtained from Foregene Biotechnology (Chengdu, China); the Taq Pro Universal SYBR qPCR Master Mix was obtained from Vazyme (Nanjing, China); the ABScript III RT Master Mix for qPCR with gDNA Remover was obtained from ABclonal (Wuhan, China).

### 2.2. Preparation, Composition, and Antioxidant Ability of RRTE

#### 2.2.1. Preparation of RRTE

We cleaned and dried RRT fruits, then removed its stems and seeds; the fruits were cut into slices to expand the surface area for drying. We used a freeze drier to remove water, and then ground it into powder by a wall-breaker to obtain RRT fruit powder. This extraction method integrated multiple references and made improvements. Ethanol extraction is commonly used as a method for extracting plant compounds. According to the studies by Armita Farid [19] and Juan Zhang [20], we know that a simple ethanol extraction can yield crude extracts containing polyphenols, flavonoids, and terpenoids from plants. Additionally, it has been reported that further enrichment can be achieved by using an alkaline solution followed by acid precipitation [21,22,23]. This may be based on the chemical structural characteristics of the target compounds, where polyphenols possess phenolic hydroxyl groups, and flavonoids and terpenes have acidic functional groups. Alkaline treatment can react with the corresponding functional groups, increasing solubility, and removing alkaline-insoluble impurities, thereby enhancing the extraction efficiency. After alkaline dissolution, taking the supernatant for acid treatment can cause the target substance to form precipitates. Subsequently, target extraction is achieved by utilizing the difference in solubility through extraction with ethyl acetate, followed by vacuum concentration to obtain the extract. The specific methods are as follows: RRT powder was enzymatically digested using 80% ethanol containing cellulase (1:5 *w*/*v*, g/mL, 60 °C, 40 min); and then we added 80% ethanol (15:1 *v*/*v*) for ultrasonic extraction (30 min, 80 °C, 3 times) using a Brinell funnel for filtration, and the filtrate was combined. The filtrate was concentrated under reduced pressure, the pH of which was adjusted to 12 using 60% NaOH solution, and the supernatant was collected by centrifugation (1500 rpm, 10 min), the pH of which was adjusted to 2 using concentrated hydrochloric acid, and then extracted with ethyl acetate (material–liquid ratio of 2:1, twice) to collect the upper ethyl acetate layer and then concentrated by spinning under reduced pressure; the concentrated extract was placed in a fume hood to evaporate the ethyl acetate, and then freeze-dried to obtain RRTE. RRTE was stored in a refrigerator at −20 °C protected from light.

#### 2.2.2. Component Analysis of the RRTE

The RRTE solution (8 mg/mL) was configured using methanol and filtered with a 0.22 organic filter membrane. Referring to our previous test parameters [24], we used liquid chromatography–high-resolution mass spectrometry (LC–HR/MS) (Thermo Fisher, Germany) to determine the possible bioactive substances in RRTE. Visualization was achieved using Thermo Xcalibur Qual Browser and Origin 2022 software, matching and compositional analysis using Compound Discoverer 3.1.1.12 software in combination with mzVault database (Supplementary Table S1). We used gallic acid, rutin, and ursolic acid, respectively, as standards to determine the contents of total polyphenols, total flavonoids, and total triterpenoids in RRTE by referring to the methods of Wang [25] and Li [26], and their standard curves are shown in Supplementary Figure S1.

#### 2.2.3. Determination of the Antioxidant Capacity of RRTE

DPPH and ABTS were determined by referring to the previous methods [25]. It is worth noting that the dilution concentration of RRTE in the ABTS-scavenging assay was up to 0, 10, 20, 40, 60, 80, 100, 120, 140, 160, 180, and 200 μg/mL, and -scavenging up to 100, 200, 300, 400, 500, 600, 700, 800, 900, and 1000 μg/mL in the DPPH-scavenging assay, which confirmed after pre-experiment.

### 2.3. Protective Effect of RRTE on Damaged NCM460 Cells

#### 2.3.1. Cell Culture

The human normal colon epithelial (NCM460) cells were purchased from ATCC (the United States) and cultured in DMEM medium containing 10% serum and 1% double antibody in a cell culture incubator at 37 °C with 5% CO_2_ concentration [10].

#### 2.3.2. Cell Viability Assay

NCM460 cells were planted in 96-well plates at 5 × 10^3^ cells/well; and after wall attachment, we added RRTE at a concentration gradient of 200–500 μg/mL for 12/24 h, and then 90 mg/mL DSS solution for 12 h. After the intervention was completed, 20 μL of MTT solution at a concentration of 5 mg/mL was added; and after incubation for 2–4 h, the solution in the wells was aspirated, DMSO solution (150 μL/well) was added and the solution was shaken for 15 min to dissolve the blue-violet crystals at the bottom, and the absorbance at 570 nm was determined [10].

#### 2.3.3. Cell Morphology Observation

NCM460 cells were planted in 6-well plates at 1.5 × 10^3^/well, and after the cells adhered to the wall, a 200–400 μg/mL concentration gradient of RRTE was added and the cells were left for 12 h, followed by the addition of 90 mg/mL DSS solution for 12 h, and then the cells were placed under a white-light microscope for observation and photographed.

#### 2.3.4. Intracellular Reactive Oxygen Species (ROS) Expression Assay

The intervention was performed in the same way as described in 2.3.3. After the intervention, we removed the cell culture medium, washed the cells three times with 2 mL PBS, digested and collected cells with EDTA-free trypsin, centrifuged the cells (800 r/3 min), and then washed the cells three times with 2 mL PBS. We added 1 mL of 2,7-dichlorofluor-escein diacetate (DCFH-DA) diluted to 10 μmol/L with serum-free culture medium, and incubated the cells in a 37 ℃ cell culture box for 30 min (gently shaking every 10 min). We washed the cells three times with serum-free cell culture medium to fully remove DCFH-DA that has not entered the cells. We used flow cytometry (FCM, BD FACSVerse) to detect fluorescence intensity, avoided light exposure throughout the process. We selected the FITC channel and collected the cells at a moderate flow rate. When 10,000 cell data were collected in the designated area, the collection was terminated to ensure that the number of cells in the designated area is constant during each collection. The statistical data of ROS expression are based on the area of fluorescence accumulation in the same size gate, which is the same as our previous method [10].

### 2.4. The Effect and Mechanism of RRT in Alleviating Ulcerative Colitis In Vivo

#### 2.4.1. Establishment of the Animal Model

Male mice (C57BL6/N, 6–8 weeks old, 19–21 g weight) used in this study were purchased from Zhejiang Viton Lihua Laboratory Animal Technology Co. Ltd. and kept in the Animal SPF Experiment Center of West China School of Public Health, Sichuan University, at 25 °C with 40–60% humidity, 12 h of light/dark cycle, and free access to drinking water and food. The animal experiment protocol was approved by the Animal Ethics Committee of West China School of Public Health, Sichuan University (Gw112022104). The mice were randomly divided into 5 groups, i.e., the control group (Group C, *n* = 6), the model group (Group M, *n* = 8), the RRTE-L group (RRTE 300 mg/kg·bw, Group L, *n* = 8), the RRTE-H group (RRTE 500 mg/kg·bw, Group H, *n* = 8), and the RRTE group (RRTE 500 mg/kg·bw, Group R, *n* = 8). After 14 d of acclimatization feeding, mice in Groups L, H, and R were gavaged with 200 μL of the corresponding dose of RRTE solution, and mice in Groups C and M were gavaged with the same volume of saline for 21 days. On day 15th, the drinking water was replaced with 3% DSS solution (except for Groups C and R); the intervention was continued for 7 days. The DSS solution was prepared with sterile water; the RRTE solution was prepared with saline. The experimental design was based on previous literature [27,28].

#### 2.4.2. Evaluation of Colonic Mechanical Barrier Damage and SYMPTOMs of Ulcerative Colitis in Mice

Mice were continuously weighed during the period and scored according to previous DAI criteria [10]. After the mice were euthanized, the colonic tissues were collected, and their lengths were measured and photographed for documentation. A sample of 1–2 cm of colon tissue was taken and fixed in 10% paraformaldehyde; hematoxylin-eosin staining (HE) was used to assess the pathological changes in the colon tissue, followed by pathological scoring [29]. The expression of the tight junction protein ZO-1 in the colon tissue was detected by IHC.

#### 2.4.3. qPCR Analysis

The colon tissues were grinded with liquid nitrogen, the mRNA from the tissues was extracted by a RNA extraction kit, and its concentration was measured. cDNA was obtained using a reverse transcription kit, real-time fluorescence quantitative PCR was performed using SYBR Green, and 2^−ΔΔCT^ was used to analyze the expression of the gene relative to GAPDH. Primers were synthesized by Shanghai Bioengineering (China), and the primer list is shown in Supplementary Table S2.

#### 2.4.4. Gut Microbiota Analysis

Collecting mice feces for the extraction of fecal DNA, quantification of the DNA by Nanodrop, detection of the quality of the DNA extraction by 1.2% agarose gel electrophoresis, and PCR amplification was performed. The TruSeq Nano DNA LT Library Prep Kit from Illumina company was used to prepare the sequencing library. The library was subjected to quality inspection on the Agilent Bioanalyzer before use; after quality inspection, the library was quantified using the Quant-IT PicoGreen dsDNA Assay Kit on the Promega QuantiFluor fluorescence quantification system (the library concentration should be above 2nM). Gradient dilution libraries were denatured to single-stranded using NaOH and then up-sequenced. Sequence denoising or OTU clustering was performed according to the QIIME2 dada2 analysis process or the analysis process of QIIME2 (2019.4; Script modified by Suzhou PANOMIX Biomedical Tech Co., LTD, chengdu, China); the alpha diversity level of the samples was assessed based on the distribution of ASV/OUT in different samples, and various modeling tools were used in combination with statistical tests to measure differences in species abundance among different samples and to find marker species.

#### 2.4.5. Transcriptomics Sequencing Analysis

We collected approximately 60 mg of mouse colon tissue and immediately froze it in liquid nitrogen; we tried our best to ensure that they were taken from the same part of the distal colon. Three samples were selected from Group C, Group M, and Group H for RNA sequencing. Total RNA from colon tissues was extracted and tested for concentration and purity; mRNA was purified to synthesize cDNA to construct libraries, and then conduct quality inspection; these libraries were subjected to paired-end (PE) sequencing using Next-Generation Sequencing (NGS) based on the Illumina sequencing platform. Filtering of the sequencing data was performed using Cutadapt to remove sequences with junctions at the 3’ end and to remove reads with average quality scores lower than Q20 to assess the sequencing quality. The filtered reads were compared to the reference genome using the HISAT2 software (2.1.0); count the expression of each sample to perform the PCA principal component analysis. Using DESeq for differential analysis of gene expression, the conditions for screening differentially expressed genes were as follows: expression difference multiple |log2FoldChange| >1, significance *p*-value < 0.05; draw a volcano map of differentially expressed genes by R language ggplots2 software package. The Euclidean method was used to calculate the distances and the hierarchical clustering longest distance method (Complete Linkage) was used for clustering. We conducted KEGG pathway significance enrichment analysis and GSEA on differentially expressed genes.

### 2.5. Data Statistics and Analysis

Data analysis and plotting were conducted using Excel, GraphPadrism 9, and SPSS 26. Data were presented as the mean ± SEM (Standard Error of Mean), and the significance of the differences between the groups was analyzed by one-way ANOVA followed by LSD test using SPSS 26 software; * *p* < 0.05, ** *p* < 0.01 *** *p* < 0.001 vs. Group C. # *p* < 0.05, ## *p* < 0.01 ### *p* < 0.001 vs. Group M.

## 3. Results

### 3.1. RRTE Compositional Analysis and Antioxidant Capacity Determination

LC–HR/MS analysis results revealed that 72 substances may exist in RRTE (Supplementary Figure S2, Supplementary Table S1), including organic acids, phenolics, flavonoids, triterpenoids and amino acids. Among them were catechin, chlorogenic acid, and other phenolic substances; rutin, quercetin, dihydrokaempferol, eupafolin, and other flavonoids; ursolic acid, oleanolic acid, rosamultin, and other triterpenoids. The total polyphenol, total flavonoid, and total triterpenoid contents of RRTE were 171.99 ± 9.02, 453.69 ± 6.66, and 237.05 ± 21.46 mg/g, respectively; while the IC_50_ values in DPPH and ABTS radical-scavenging activity were 0.43 ± 0.018 mg/mL and 0.078 ± 0.0055 mg/mL, respectively (Table 1), which indicated that we can successfully enrich the active components and retain their excellent antioxidant capacity of RRT by this extraction method.

### 3.2. RRTE Could Prevent NCM460 Cells from DSS-Induced Injury

**Figure 1:**
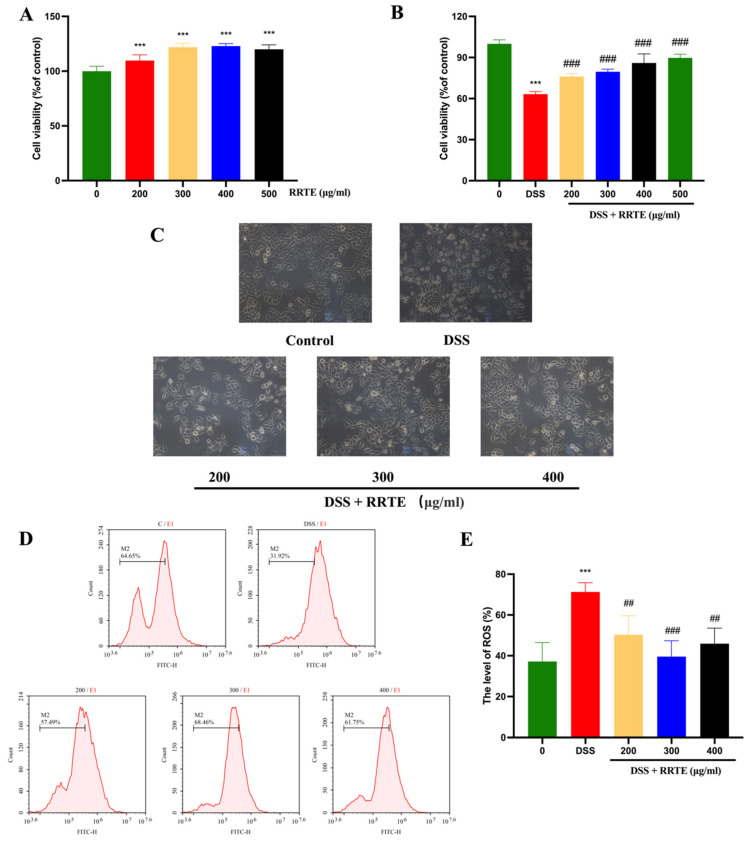
RRTE prevented the damage of viability, morphological, and the ROS levels of NCM460 cells after DSS damage. (**A**) Effect of RRTE on NCM460 cells viability. (**B**,**C**) The preventive effect of RRTE on the cell viability and morphological damage of NCM460 cells induced by DSS. (**D**,**E**) RRTE reduced intracellular ROS levels. Data are presented as the mean ± SEM of at least three independent experiments. *** *p* < 0.001 vs. control group (0). ## *p* < 0.01, ### *p* < 0.001 vs. DSS group.

DSS-induced NCM460 cells can mimic UC in vitro [30,31], RRTE at 200–500 μg/mL could extremely significantly restore the DSSinduced decrease in cell viability (Figure 1A, B); in this dose, RRTE did not damage NCM460 cells. Under the whitelight microscope, we can observe that after DSS-induced the cells showed dead cell morphology such as rounding, crumpling, accompanied by floating dead cells, and lost the morphological characteristics of epithelial cells. After RRTE intervention, there were fewer floating dead cells and cell morphology was restored (Figure 1C). Oxidative stress promotes UC progression; excessive release of ROS can disrupt redox homeostasis, and promote inflammatory progression and oxidative damage thereby accelerating apoptosis in colon cells [31]. RRTE dramatically reduced the DSS-induced increase in ROS levels against oxidative damage (Figure 1D,E). These results indicate that RRTE can prevent cell damage induced by DSS in vitro.

### 3.3. RRTE Relief DSS-Induced Ulcerative Colitis in Mice

#### 3.3.1. RRTE Alleviates Colitis Symptoms in UC Mice

**Figure 2:**
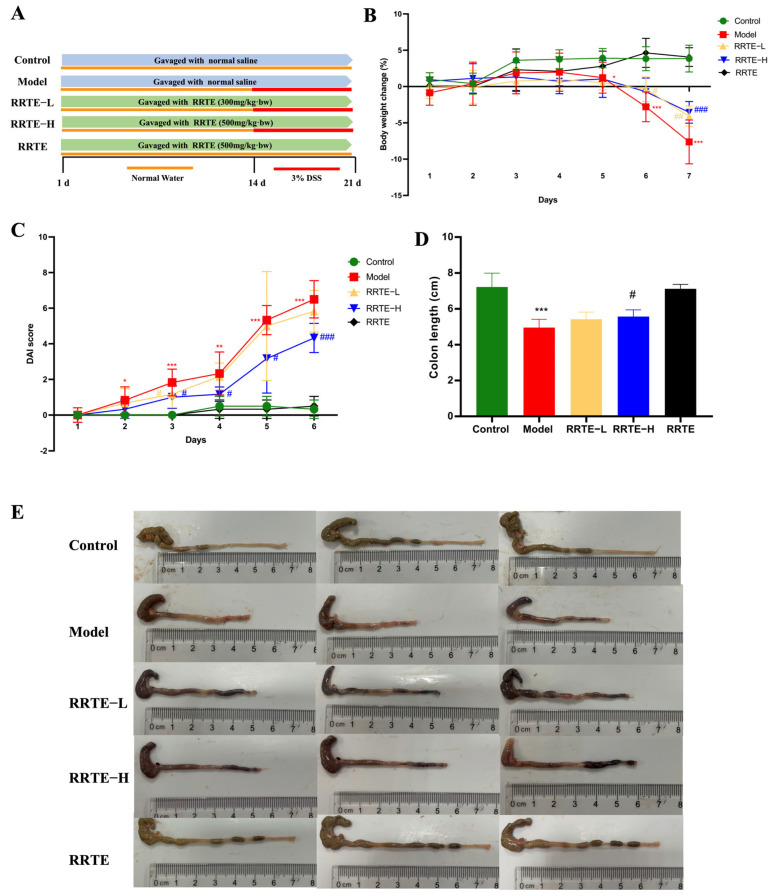
RRTE can alleviate the symptoms of ulcerative colitis in mice. (**A**) The design of animal experiment; (**B**) changes in body weight; (**C**) DAI score; (**D**,**E**) colon length and images. Data are presented as the mean ± SEM (*n* = 6). * *p* < 0.05, ** *p* < 0.01, *** *p* < 0.001 vs. Group C. # *p* < 0.05, ## *p* < 0.01, ### *p* < 0.001 vs. Group M.

The experimental design scheme is shown in Figure 2A. In this study, we further investigate the relieving action of RRTE in UC mice. During the seven days of adding 3% DSS drinking water, the weight of mice in Groups M, L, and H declined constantly, while the loss rate of weight in the L and H groups was lower than that of Group M (Figure 2B); DAI scores revealed that RRTE lowered DAI scores and effectively alleviated diarrhea and blood in stool in mice (Figure 2C); meanwhile, RRTE alleviated the shortening of colon length in UC mice, especially the high dose was better than the low-dose (Figure 2D,E). These results suggest that RRTE could alleviate colitis symptoms in UC mice.

#### 3.3.2. RRTE Protects the Colon Mucosa Mechanical Barrier

**Figure 3:**
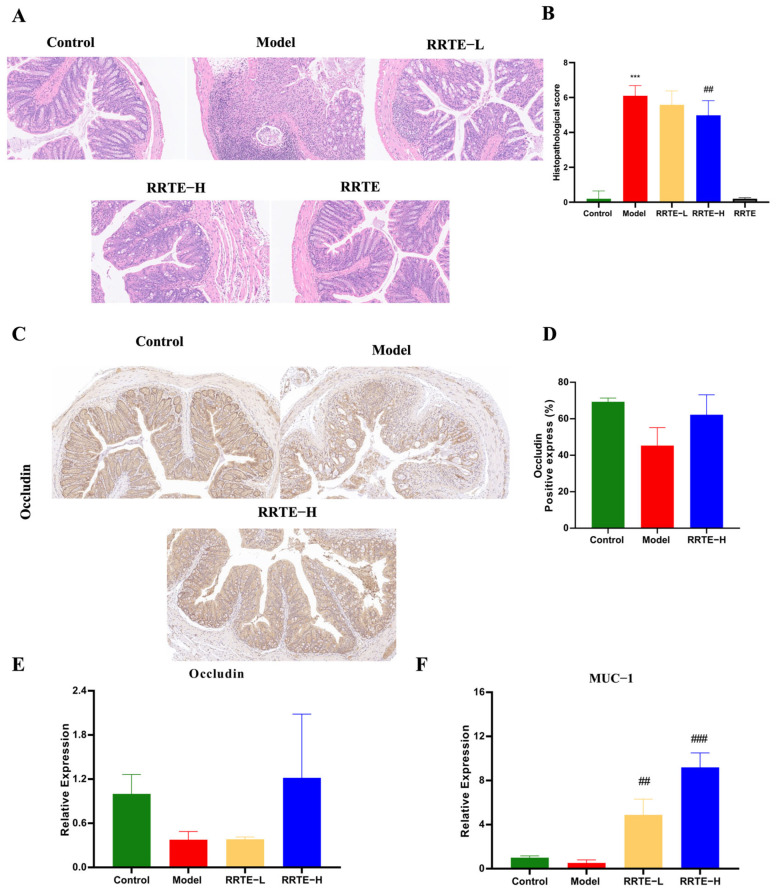
RRTE can protect the colonic mechanical barrier in UC mice. (**A**,**B**) H&E staining and pathological tissue scoring (10×). (**C**,**D**) Immunohistochemical staining of occludin in colon tissue (20×), and the percentage of positive cells for occludin. (**E**,**F**) mRNA expression level of occludin and MUC-1. Data are presented as the mean ± SEM (*n* = 3). *** *p* < 0.001 vs. Group C. ## *p* < 0.01, ### *p* < 0.001 vs. Group M.

The colon mucosa mechanical barrier is critical in the development of UC. HE staining results showed that Group M lost the normal tissue structure of the colon, the crypt structure was destroyed, and accompanied by severe inflammatory infiltration; fortunately, high-dose RRTE intervention can significantly reduce pathological scores and alleviate pathological damage (Figure 3A,B). Furthermore, IHC data demonstrated that RRTE intervention increased the expression of the tight junction protein occludin, but there was no significant difference (Figure 3C,D). We further used qPCR to detect the mRNA expression of tight junction protein occludin, and mucin MUC1 (Figure 3E,F). These results showed that RRTE could increase the mRNA expression levels of occludin and MUC1, especially at high doses, which significantly increased MUC1 expression levels. These findings suggest that RRTE can protect the colon mucosa mechanical barrier.

#### 3.3.3. RRTE Reduces Colonic Mucosal Inflammation

**Figure 4:**
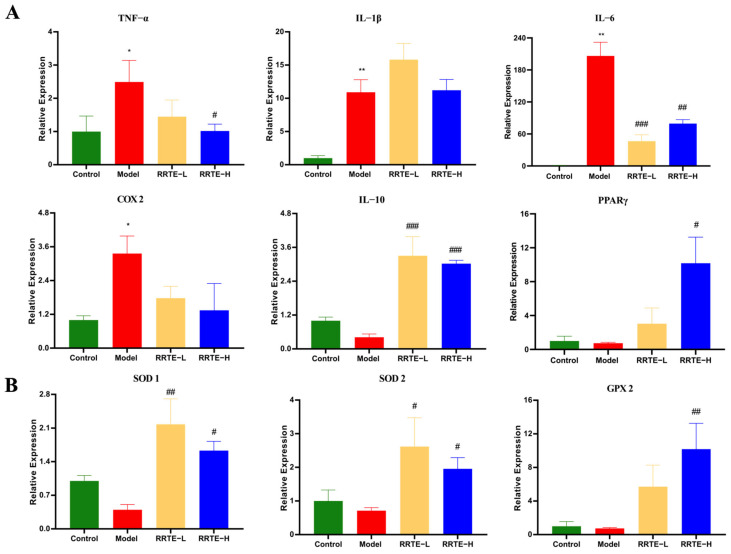
RRTE can alleviate inflammation and oxidative damage in the colon. (**A**) mRNA expression level of TNF-α, IL-6, IL-1β, COX 2, IL-10 and PPARγ. (**B**) mRNA expression level of SOD1, SOD2, and GPX 2. Data are presented as the mean ± SEM (*n* = 4). * *p* < 0.05, ** *p* < 0.01 vs. Group C. # *p* < 0.05, ## *p* < 0.01, ### *p* < 0.001 vs. Group M.

The inflammatory infiltration of colon mucosa is one of the main pathological features of UC [32], and it is also one of the risk factors for the damage of colon mucosal barrier [33]. We examined the mRNA expression levels of relevant inflammatory factors and pro-inflammatory factors; the results showed that DSS-induced up-regulated the expression of TNF-α, IL-6, IL-1β, and COX 2, and down-regulated the expression of IL-10 and PPARγ, which could be reversed by RRTE intervention (Figure 4A). These data suggest that the protective effect of RRTE in UC is related to its modulation of inflammation.

#### 3.3.4. RRTE Reduces the Oxidative Stress of the Colon Mucosa

Furthermore, oxidative stress is also an important risk factor in the progression of UC disease, as it not only exacerbates damage to the intestinal mucosal barrier but also promotes the development of inflammation [34]. We detected the mRNA expression levels of three important antioxidant enzymes through qPCR to evaluate the effect of RRTE on DSS-induced colonic oxidative stress in mice. The expression of SOD1, SOD2, and GPX2 showed a decreasing trend after DSS was induced, and RRTE intervention could eliminate this effect and maintain its expression at high levels (Figure 4B). This indicates that RRTE can effectively enhance the antioxidant level in the body to alleviate UC.

#### 3.3.5. RRTE Can Regulate the Gut Microbiota of UCMice

**Figure 5:**
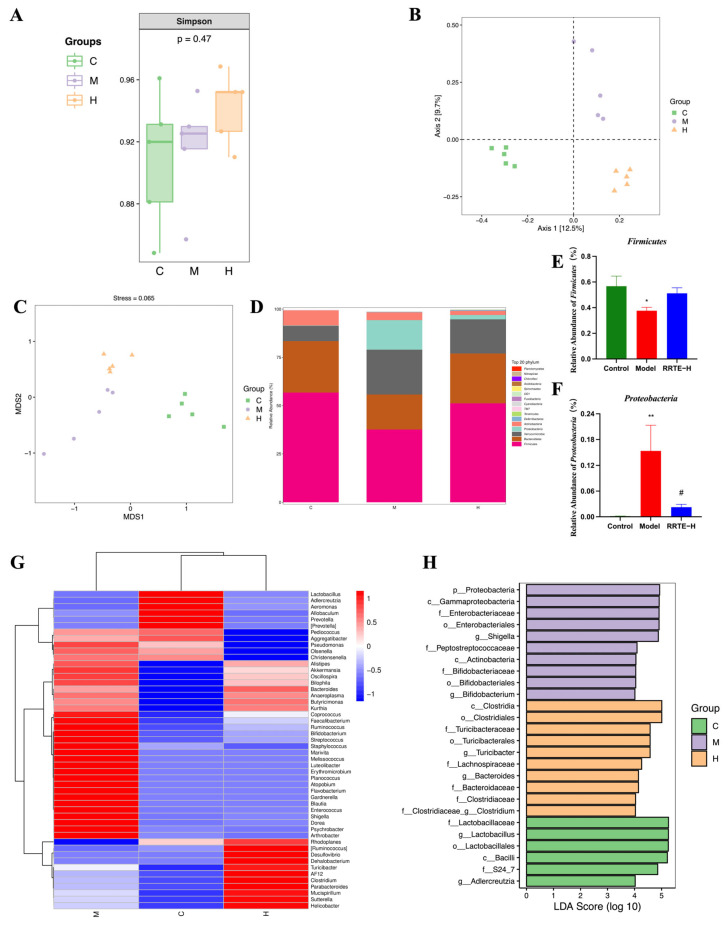
RRTE can regulate the gut microbiota. (**A**) Simpson index. (**B**) Principal Co-ordinate Analysis (PCoA). (**C**) non-metric multidimensional scaling (NMDS). (**D**) Microbiota compositions at the phylum level. (**E**,**F**) Abundance of *Proteobacteria* and *Firmicutes* in each group. (**G**) Heat map of changes in gut microbiota at the genus level for groups. (**H**) Linear discriminant analysis with LDA score greater than 4. Data are presented as the mean ± SEM (*n* = 5). * *p* < 0.05, ** *p* < 0.01 vs. Group C. # *p* < 0.05 vs. Group M.

The gut microbiota is a complex microbial ecology, and the microorganisms and metabolites produced by them have a substantial impact on the host’s health. UC patients generally suffer from gut microbiota imbalance [35,36]; current research has found that modulating the composition of gut microbes can reduce inflammation and restore the intestinal mucosal barrier in patients [37]. We conducted 16S rRNA sequencing on mouse feces to see if RRTE can modulate gut microbiota disorders. The abundance and diversity of gut microbiota were evaluated using the Simpson index to represent alpha diversity. The results showed that there were no significant differences among the groups, but it is worth noting that the values of Group H were slightly higher than others (Figure 5A). Non-metric Multidimensional Scale of Evaluation (NMDS) and Principal Coordinate Analysis (PCoA) were used to represent the β-diversity to observe the similarity of microbial composition. The results showed that the groups were separated from each other, but the flora within the groups were clustered together, indicating that the structure of the gut microbiota in each group had changed (Figure 5B,C). To further explore the structure of gut microbiota in each group of mice, we analyzed its specific composition. At the phylum level, compared with Group C, the abundance of *Firmicutes* significantly reduced in Group M, and the abundance of *Proteobacteria* significantly increased, while RRTE can reverse this change, especially in Proteobacteria (Figure 5D–F). At the genus level, the heatmap of species abundance showed that compared to Group C, the abundance of pathogenic bacteria such as *Staphylococcus*, *Shigella*, *Enterococcus*, *Gardnerella*, and *Flavobacterium* in Group M was increased. The RRTE intervention in Group H reduced the abundance of these pathogenic bacteria while increasing the abundance of beneficial bacteria such as *Ruminococcus*, *Turicibacter*, and *Parabacteroides*; and the UPGMA clustering results showed that the microbial compositions of Groups H and C were more similar (Figure 5G). Using linear discriminant analysis (LDA) to identify the dominant species of each group, we found that most of the dominant species in Group M were *Proteobacteria*, while in Group H were *Firmicutes* (Figure 5H). The above results indicate that RRTE can lower the abundance of pathogenic bacteria while increasing the abundance of beneficial bacteria to regulate the gut microbiota disorders caused by DSS.

#### 3.3.6. Data Analysis on the Mouse Colon Transcriptome

**Figure 6:**
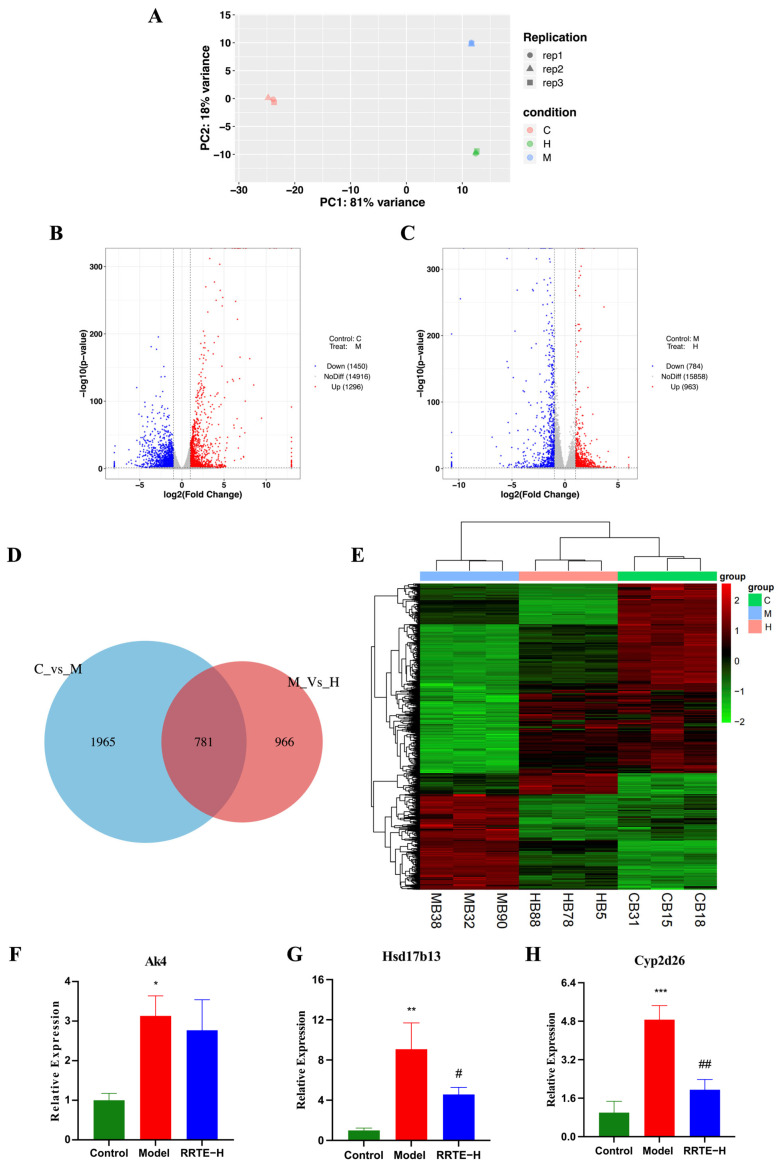
The effect of RRTE at the genetic level. (**A**) Principal component analysis (PCA) diagram. (**B**,**C**) Volcano plot comparison between Group C and Group M, Group M and Group H. (**D**,**E**) Venn and heatmaps of differentially expressed genes shared by C vs. M and M vs. H. (**F**–**H**) mRNA expression level of Akt4, Hsd17b13, Cyp2d26. Data are presented as the mean ± SEM (*n* = 3). * *p* < 0.05, ** *p* < 0.01, *** *p* < 0.001 vs. Group C. # *p* < 0.05, ## *p* < 0.01vs. Group M.

To further reveal the mechanism of RRTE in alleviating UC, we conducted transcriptomic analysis on the colon of mice in Groups C, M, and H. In sequencing, each sample can contribute an average of 6.29 G base numbers, and the Q30 base percentage of the sample is above 92.9% (in Supplementary Table S3), indicating that the transcriptome data are rich and of high quality. PCA analysis showed that there were differences in gene expression among different groups (Figure 6A). The sequencing results showed that compared with Group C, 1296 genes were up-regulated and 1450 genes were down-regulated in Group M, resulting in a total of 2746 differentially expressed genes (DEGs) (P < 0.05) (Figure 6B). Compared with Group M, 963 genes were up-regulated and 784 genes were down-regulated in Group H, resulting in a total of 1747 DEGs (P < 0.05) (Figure 6C). There are 781 DEGs in C vs. M and M vs. H (Figure 6D); the heatmap of the shared DEGs between C vs. M and M vs. H showed that RRTE can restore the expression levels of some genes in Group M to levels comparable to those in Group C; and the results of cluster analysis showed that the DEGs expression of Group C and Group H were more similar (Figure 6E). Using qPCR to validate some DEGs, we observed that Adenylate Kinase 4 (Ak4), an inflammation-related gene, and hydroxysteroid 17β-dehydrogenase 13 (HSD17B13), cytochrome P450 enzymes-Cyp2d26, liver injury-related genes, were both highly expressed in Group M while the RRTE could mitigate this alteration (Figure 6F–H), which was consistent with the results of the transcriptomic analyses, proving the reliability of the transcriptomic results. In addition, the increased expression levels of HSD17B13 and Cyp2d26 in Group M demonstrate that UC mice not only exhibit intestinal damage but also cause liver damage, which is consistent with previous studies [38].

**Figure 7:**
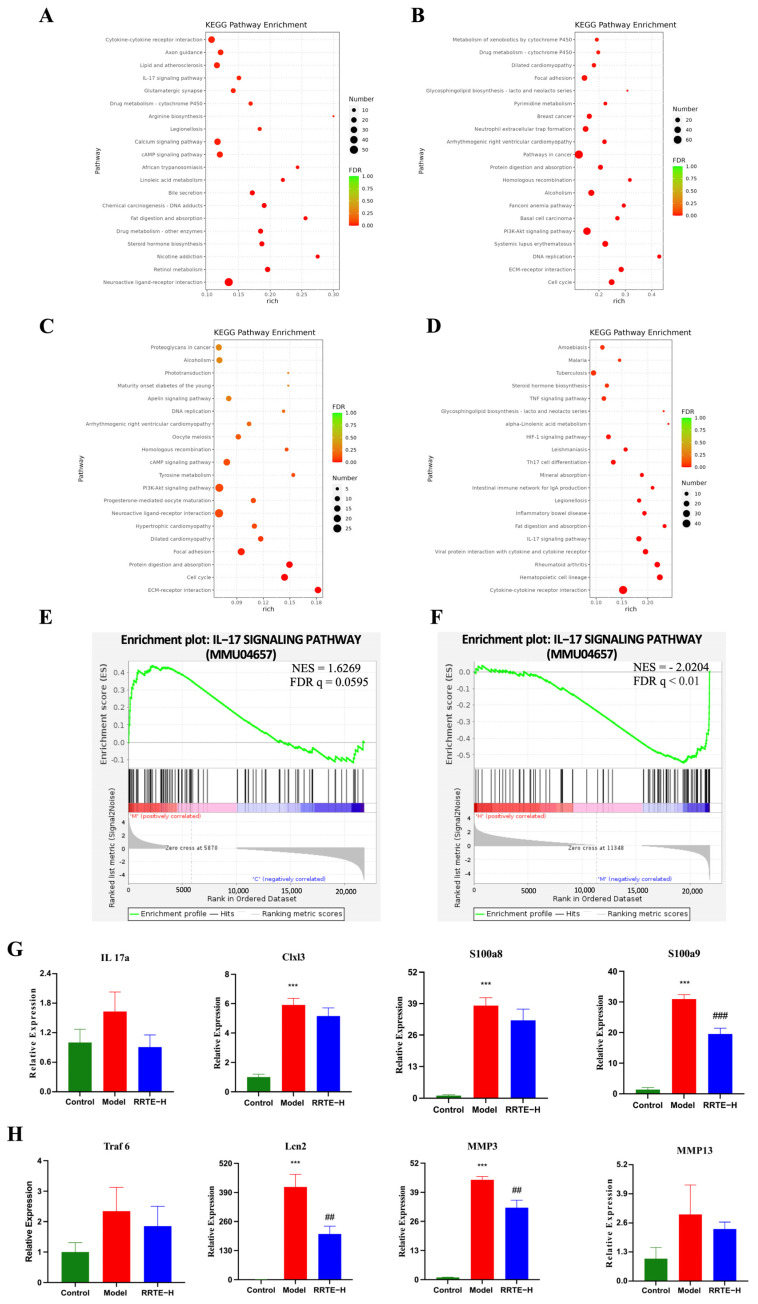
KEGG and GSEA enrichment analysis, and qPCR validation. (**A**) KEGG pathways of up-regulated differentially expressed genes (DEGs) in C vs. M. (**B**) KEGG pathways of down-regulated DEGs in C vs. M. (**C**) KEGG pathways of up-regulated DEGs in M vs. H. (**D**) KEGG pathways of down-regulated DEGs in M vs. H. (**E**) Gene-set-enrichment analysis (GSEA) of C vs. M DEGs in the IL-17 signaling pathway. (**F**) Gene-set-enrichment analysis (GSEA) of M vs. H DEGs in the IL-17 signaling pathway. (**G**,**H**) mRNA expression level of IL-17a, Clxl3, S100a9, S100a8, Traf6, Lcn2, MMP3 and MMP13. Data are presented as the mean ± SEM (n = 5). *** *p* < 0.001 vs. Group C. ## *p* < 0.01, ### *p* < 0.001 vs. Group M.

KEGG enrichment analysis showed that compared with Group C, the up-regulated DEGs in Group M were enriched in immune-related pathways such as cytokine-cytokine receptor interaction, the IL-17 signaling pathway, the CAMP signaling pathway, and were involved in chemical carcinogenesis-DNA adducts, drug metabolism, Lipid and atherosclerosis, linoleic acid metabolism, and Legionellosis (Figure 7A); the down-regulated DEGs are also enriched in drug metabolism, in addition to the PI3K-Akt signaling pathway, cell cycle, cardiomyopathy, cancer, and other related pathways (Figure 7B). The above results suggest that the ingestion of DSS may trigger inflammatory response and release inflammatory mediators, which in turn affects the expression and activity of drug metabolism-related enzymes such as drug metabolism-cytochrome P450 in the liver, which is consistent with the results of previous studies [39]; in addition to the persistent inflammatory state can cause damage to DNA can also affect the proliferation of cells, which can provide favorable conditions for the development of tumors [40], and can also be involved in the progression of other diseases. Compared with Group M, the down-regulated DEGs in Group H were not only enriched in the TNF, IL-17, TH17, HIF signaling pathways, but also involved in inflammatory bowel disease and Intestinal immune network for IgA production (Figure 7D). The up-regulated DEGs KEGG was enriched in cell cycle, cancer, PI3K-Akt and other pathways (Figure 7C). This suggests that RRTE intervention may regulate inflammation-related pathways and inhibit the progression of UC and other related diseases.

Subsequently, gene-set-enrichment analysis (GSEA) was used to further reveal the potential molecular mechanism of RRTE in UC. Compared with Group C and H, Group M was enriched in the IL-17 signaling pathway and was significantly up-regulated (Figure 7E,F). Using qPCR to validate 12 core shared genes identified by GSEA analysis in the IL-17 signaling pathway, including Clxl3, IL-17a, and Mapk14. The results showed that the expression of Cxcl3, IL-1β, IL-17a, IL-6, MMP14, MMP3, S100a9, S100a8, and Traf6 of the IL17 signaling pathway could be up-regulated after DSS induction, whereas the expression of these genes could be down-regulated by the RRTE (Figure 7G,H). Therefore, we concluded that RRTE could regulate the IL-17 signaling pathway to alleviate UC.

## 4. Discussion

The clinical symptoms of UC and the side effects associated with its treatment seriously affect patients’ quality of life and exacerbate the disease burden. At present, the incidence rate and prevalence of UC are increasing worldwide; UC has become a global public health problem. Anti-inflammation, anti-oxidant, and regulation of gut microbiota are the main strategies for treating UC. However, the side effects and costs of these drugs are the main issues faced by UC treatment. High efficiency, low toxicity, and affordable natural products are candidates for UC therapeutic supplements. RRT has a long history of consumption and medicinal use in southwestern China, and is rich in active ingredients with anti-inflammatory and anti-oxidant properties. In this study, RRTE effectively enriched the active components in RRT fruit. Its total phenolic, total flavonoid and total triterpenoid contents were 5.12, 9.07, and 22.47 times higher than those in each gram of RRT fruit, respectively [41]. These substances endow RRTE with strong anti-oxidant capacity.

Firstly, we investigated the preventive effect of RRTE on DSS-induced damage to NCM460 cells. The results showed that RRTE can restore the cell viability and morphological changes induced by DSS. In addition, we observed that intracellular ROS levels increased after DSS was induced while RRTE can restore them to normal levels. These indicate that RRTE can protect NCM460 cells from damage caused by DSS and imply its potential in UC treatment. Subsequently, we established the UC mice model to investigate the protective effect of RRTE in UC. Using chemical inducer DSS to establish the mice ulcerative colitis model is currently a recognized method, with a high success rate and strong stability and reproducibility, which can enable mice to present UC-related clinical symptoms and pathological features [27]. After DSS was induced the weight of Group M mice gradually decreased, with symptoms of diarrhea and bloody stools, the DAI score gradually increased, and the colon length was significantly lower than Group C, which indicated that the UC model was successfully established; however, RRTE can alleviate these symptoms.

The major pathogenic feature of UC is the impaired colonic mucosal barrier [42]; therefore, protecting the colonic mucosal barrier is the fundamental goal of UC treatment. The intestinal mechanical barrier is an important component of the colonic mucosal barrier, consisting of a mucous layer, epithelial cells, and tight junctions (TJs). Once the barrier structure is damaged, it will increase intestinal permeability and cannot resist the invasion of pathogenic bacteria and harmful substances, thus triggering intestinal inflammation [43]. The HE results showed that RRTE can protect the normal structure of colon tissue and reduce inflammatory infiltration. The transmembrane protein occludin and mucin MUC1 are important components of the TJs and mucus layer, respectively [44], which together maintain the intestinal mechanical barrier; aberrant expression of these protein expressions can increase intestinal permeability and promotes intestinal inflammation [45]. RRTE can increase the expression of occludin and MUC1, suggesting that RRTE can play a protective role in UC by protecting the intestinal mechanical barrier.

Another major pathologic feature of UC is persistent intestinal inflammation. After the mechanical barrier is damaged, it would activate the innate immune response, and overactivated immune cells such as macrophages would release proinflammatory factors such as TNF-α, IL-6 and IL-1β [46], which is consistent with what has been observed in patients with UC. These inflammatory factors can regulate the proliferation and differentiation of T cells [47], activate the inflammatory signaling pathway, promote the sustained development of inflammation [43,48], and increase intestinal permeability, which could cause a vicious cycle and accelerate the progression of UC [49]; moreover, persistent inflammatory damage can also increase the risk of cancer [50]. Therefore, these inflammatory factors are considered as indicators to measure the severity of UC. To delay disease progression and reduce the risk of colorectal cancer, anti-inflammatory therapy is the main goal of UC treatment. Peroxisome proliferator-activated receptor γ (PPARγ), a key factor in the regulation of macrophage activation and NF-κB, inhibits the release of pro-inflammatory factors (IL-6 and IL-1β and TNF-α) and inflammatory mediators (COX2, etc.) to reduce inflammation [51]; so PPARγ is a potential therapeutic target for UC, the first-line drugs 5-aminosalicylic acid (5-ASA) and rosiglitazone are PPAR-γ agonists [52]. In this study, we observed that the mRNA levels of IL-6, IL-1β, and TNF-α were elevated after DSS was inducedwhich was consistent with the findings of Xiaotian Xu et al. in UC mice [33]; whereas RRTE can decrease the expression of IL-6, TNF-α, and COX2, increase the expression of the anti-inflammatory factor IL-10, which may be related to the increased expression of PPAR-γ after the intervention. These demonstrate that RRTE has anti-inflammatory effects.

Inflammation and oxidative stress are closely linked. Inflammation triggers the production of free radicals, so intense inflammation will inevitably disrupt the redox balance of the body [53]. Excessive accumulation of ROS can cause oxidative damage to intracellular lipids, proteins, and DNA, which can also activate the pro-inflammatory signaling pathway to induce the release of pro-inflammatory cytokines thereby exacerbating inflammation [54]; the two are mutually reinforcing, and both would cause damage to the intestinal mechanical barrier and increase intestinal permeability, oxidative stress is an important factor in promoting the development of UC [55,56]. Therefore, alleviating oxidative stress and regulating the redox balance is a promising therapy for UC. Saffron supplementation helped increase antioxidant capacity in UC patients to reduce oxidative stress and inflammation, and decrease disease severity [57]. The Gegen Qinlian decoction (MGQD) restores the expression levels of SOD, GSH, and CAT in UC mice to attenuate DSS-induced oxidative stress injury [58]. In our study, we found that RRTE can restore the intracellular ROS levels to normal in vitro, which can also promote the expression levels of ROS-scavenging enzymes SOD1, SOD2, and GPX2, thus RRTE could exert antioxidant effects in UC mice.

Disturbance of gut microbiota is one of the characteristics of UC, the gut microbiota is involved in the energy metabolism of nutrients, and in immune metabolism to maintain host health [59]. Pathogenic bacteria and the toxins they produced can activate the intestinal immune response and inflammatory signaling pathways, such as the NF-κB pathway, to promote the release of inflammatory factors and disrupt the integrity of the intestinal barrier to cause local or systemic inflammation [60,61]. The composition of the gut microbiota of patients with UC differs from that of the healthy population, manifested as a decrease in the number of *Firmicutes*, and an increase in the number of *Bacteroides*, *Proteobacteria*, and anaerobic microbiota [62,63]. However, the causal relationship between ulcerative colitis and gut microbiota dysbiosis is still unknown, but it is undeniable that improving the composition of gut microbiota is beneficial for repairing the colonic mucosal barrier and enhancing local and systemic immunity, which has gradually become a new treatment for UC. Our study found that RRTE can inhibit the abundance of pathogenic bacteria such as *Staphylococcus* [64], *Shigella* [65], and *Enterococcus* [45]; at the same time, increase the abundance of *Ruminococcus* and *Turicibacter*, which are associated with the production of short-chain fatty acids [66,67], as well as *Parabacteroides*, which have protective effects in UC and are considered the next generation of probiotics. In addition, LDA analysis results showed that g_*Turicibacter* is the dominant bacterial group in Group H. Relevant studies have shown that its abundance was negatively correlated with the expression of IL-1β, IL-6, and TNF-α. These indicate that RRTE can alleviate the imbalance of gut microbiota induced by DSS.

In addition, KEGG results of transcriptomics showed that RRTE intervention could participate in the regulation of inflammation-related signaling pathways such as the TNF, IL-17, TH17, and HIF signaling pathways. GSEA combined with qPCR confirmed that RRTE is involved in regulating the IL-17 signaling pathway and reducing the expression of pro-inflammatory factors (IL-1β, IL-17a, IL-6), Clxl3, S100a9, Traf 6, MMP14, MMP3. The chemokine family (CXCL) and the S100 family can recruit innate immune cells such as granulocytes to enter the inflammatory site; serving as a pro-inflammatory mediator S100a8 and S100a9 is also participating in the inflammatory signaling cascade; elevated expression of CXCL3, S100a9, and S100a8 has been observed in human colon polyps and tumors, as well as in mouse UC colon tissue [68]; anti-S100a9 treatment can inhibit inflammatory symptoms related to IBD and delay the development of colon cancer [69]. Additionally, Traf 6 of the tumor necrosis factor receptor-related factor family (TRAF) can activate NF-κB; Liu [47] demonstrated that Oxysophocarpine (OSC) alleviates oxidative stress and inflammation in UC mice by inhibiting the Traf 6 pathway. Lcn 2 may be related to the progression of ulcerative colitis-associated carcinogenesis, and can serve as a new biomarker for tumor detection, which helps in early diagnosis [70]; moreover, Lcn 2 is a key factor in the regulation of ferroptosis in UC, silencing of Lcn 2 suppressed ferroptosis events [71]. The family of matrix metalloproteinases (MMPs) is also involved in various physiological processes such as inflammation, cancer, and wound healing; among them, MMP14 and MMP3 are involved in tumor progression [72,73]. RRTE can regulate the IL-17 signaling pathway and reduce the expression of these factors.

RRTE exhibits superior relieving effects in UC. The biological activity of the extract is closely related to their material composition. LC-HR/MS results showed that RRTE may be rich in various bioactive substances, including catechin, procyanidin [74], ursolic acid [75], chlorogenic acid [76], quercetin [77], gallic acid [78], oleanolic acid [79], and naringenin [80]. These substances have been proven to alleviate UC. Catechins and procyanidin can reduce inflammation levels in serum and colonic tissues; pharmacokinetic results indicate that they can be widely distributed in serum and tissues, and can reach diseased colonic tissues to further increase anti-UC activity [74]. Chlorogenic acid exerts therapeutic effects on UC by inhibiting the MAPK/ERK/JNK signaling pathway [76]. Gallic acid ameliorated the disease symptoms and inflammatory damage in UC mice by inhibiting the NLRP3 inflammasome [78]. Oleanolic acid modulates Th17/Treg cell balance and inhibits the NF-κB signaling pathway to protect UC mice [79]. Naringenin reduced oxidative damage and the release of inflammatory mediators in a dose-dependent manner to alleviate UC [80]. These substances may be the material basis for RRTE to alleviate DSS-induced NCM460 cell damage and UC mice. In addition, the LC-HR/MS results showed that RRTE also contains other polyphenols, flavonoids, and terpenoids, which may also be the material basis for exerting the efficacy of RRTE, but further research is needed.

In summary, this study fully utilizes the Chinese characteristic fruit resource, *Rosa roxburghii* Tratt, and focuses on the polyphenols, flavonoids, and terpenoids in it to successfully prepare the RRTE extract. It enriches the active substances in RRT and has strong antioxidant capacity. We comprehensively explored how RRTE could prevent the damage induced by DSS both in vivo and in vitro, and preliminarily explored its possible mechanism. It can protect the viability and morphology of NCM460 cells, and alleviate oxidative damage induced by DSS. In UC mice, RRTE can alleviate symptoms related to UC, protect the normal tissue structure of the colon, increase the expression of tight junction proteins and mucins, and maintain the intestinal mechanical barrier, which can also reduce the release of inflammatory factors and mediators, and increase the expression of ROS-scavenging enzymes to reduce damage to colon tissue. The transcriptome results suggest that RRTE may be involved in the regulation of multiple inflammatory signaling pathways. qPCR confirms that RRTE intervention can regulate the IL-17 signaling pathway and reduce the expression of related factors. In addition, RRTE can regulate gut microbiota disorders, increase the abundance of beneficial bacteria such as short-chain fatty acid-producing bacteria, and especially increase the level of g_*Turicibacter* that negatively correlated with the expression of inflammatory factors. Therefore, RRTE may become a potential supplement for UC treatment.

## 5. Conclusions

To sum up, our research indicates that RRTE is a *Rosa Roxburghii* Tratt fruit extract with a strong antioxidant capacity. It can alleviate the injury of NCM460 cells induced by DSS; in UC mice, RRTE can protect the intestinal mechanical barrier, alleviate inflammation and oxidative damage, and regulate the gut microbiota and the IL-17 signaling pathway to alleviate UC. Therefore, RRTE has great potential in preventing and treating UC, which provides a new strategy for developing UC treatment drugs.

## Figures and Tables

**Table 1:** The components and antioxidant capacities analysis in RRTE. Data are presented as the mean ± SEM (*n* = 3).

Total Polyphenols mg/g	Total Flavonoids mg/g	Total Triterpenoids mg/g	DPPH IC_50_ mg/mL	ABTS IC_50_ mg/mL
171.99 ± 9.02	453.69 ± 6.66	237.05 ± 21.46	0.43 ± 0.018	0.078 ± 0.0055

## Data Availability

Data will be made available on request.

## Supplementary Materials

The following supporting information can be downloaded at: https://www.mdpi.com/article/10.3390/nu15214560/s1, Figure S1: The standard curves of chlorogenic acid, ursolic acid, and rutin; Figure S2: Total ion chromatogram of RRTE; Table S1: The possible substances in RRTE; Table S2: Sequences of primers used in this study; Table S3: Offline data statistics.
