# Supplementary material for: Rosa Roxburghii Tratt Fruit Extract Prevents Dss-Induced Ulcerative Colitis in Mice by Modulating the Gut Microbiota and the IL-17 Signaling Pathway
Source: Nutrients. 2023 Oct 27;15(21):4560. doi: 10.3390/nu15214560 (PMC10650662; doi:10.3390/nu15214560)
Supplement: Supplementary file 1 [file nutrients-15-04560-s001.zip › Supplementary Figure and Table.pdf]

Supplementary Figure and Table

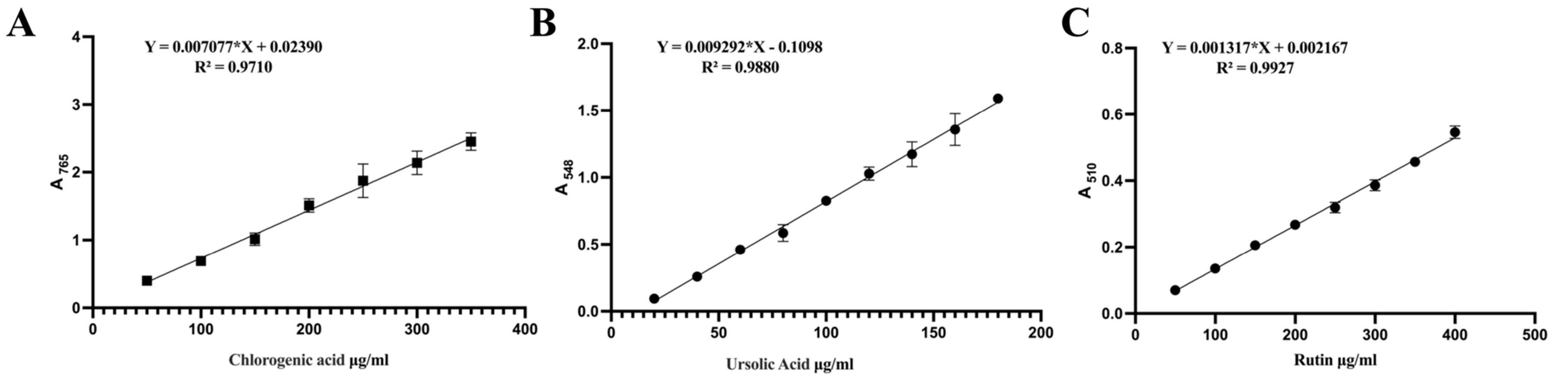

Supplementary Figure S1. The standard curves of Chlorogenic acid, Ursolic Acid, and Rutin.

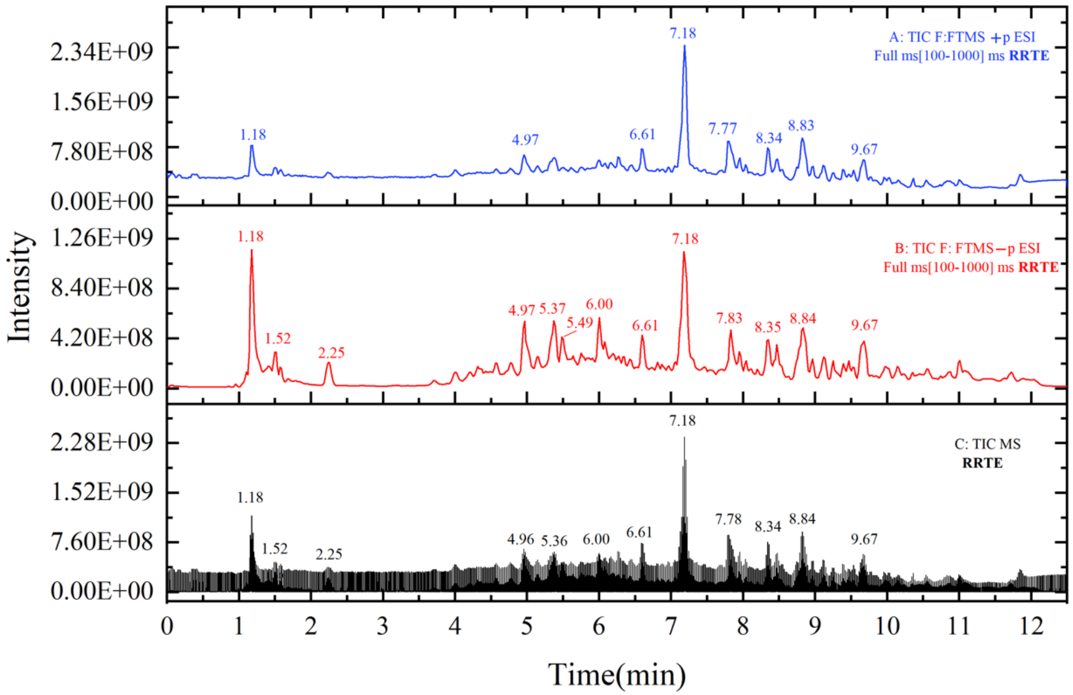

Supplementary Figure S2 Total ion chromatogram of RRTE.

Supplementary Table S1 The possible substances in RRTE

Number	Name	Formula	Molecular weight	Retention Time	Pseudomolecular ion peak	Classification
1	Arginine	$C_6H_{14}N_4O_2$	174.11158	0.939	$[M+H]^+$ 175.11877	Amino acids
2	D-FRUCTOSE	$C_6H_{12}O_6$	180.06325	1.025	$[M-H]^-$ 179.0559	Glycosides
3	L-Glutamic acid	$C_5H_9NO_4$	147.05308	1.047	$[M-COOH]^-$ 102.5488	Amino acids
4	5-Hydroxymethylfurfural	$C_6H_6O_3$	126.03165	1.066	$[M-OH]^-$ 109.02846	Furan compounds
5	2-Pyrrolidinecarboxylic acid	$C_5H_9NO_2$	133.07386	1.091	$[M+H-H_2O]^+$ 116.07056	Amino acids
6	D-(-)-Quinic acid	$C_7H_{12}O_6$	192.06324	1.108	$[M-H]^-$ 191.05608	Organic acid
7	Sucrose	$C_{12}H_{22}O_{11}$	342.11588	1.272	$[M-H]^-$ 341.1086	Glycosides
8	D-(+)-Malic acid	$C_4H_6O_5$	134.02144	1.54	$[M-H]^-$ 133.0141	Organic acid
9	Citric acid	$C_6H_8O_7$	192.02694	1.55	$[M-H]^-$ 191.0196	Organic acid
10	L-Tyrosine	$C_9H_{11}NO_3$	164.04716	1.802	$[M-COOH]^-$ 136.07558	Amino acids
11	Meglutol	$C_6H_{10}O_5$	162.05274	1.837	$[M-H]^-$ 161.04544	Amino acids
12	Gallic acid	$C_7H_6O_5$	170.02151	2.339	$[M-H]^-$ 169.01418	Polyphenols
13	l-ascorbic acid	$C_6H_8O_6$	176.03206	2.801	$[M+H]^+$ 177.03935	Vitamins
14	(-)-Gallocatechin	$C_{15}H_{14}O_7$	306.07386	3.692	$[M-H]^-$ 305.06641	Polyphenols

15	Epigallocatechin	$C_{15}H_{14}O_7$	306.07388	3.985	$[M-H]^-$ 305.0666	Polyphenols
16	Chlorogenic acid	$C_{16}H_{18}O_9$	354.09487	4.14	$[M-H]^-$ 353.08771	Polyphenols
17	L-Tryptophan	$C_{11}H_{12}N_2O_2$	204.08977	4.342	$[M-H]^-$ 203.0825	Amino acids
18	(+)-Epicatechin	$C_{15}H_{14}O_6$	290.07892	4.491	$[M+H]^+$ 291.0863	Flavanols
19	Tryptophan	$C_{11}H_{12}N_2O_2$	204.08969	4.502	$[M-H]^-$ 203.0825	Amino acids
20	Cryptochlorogenic acid	$C_{16}H_{18}O_9$	354.09494	4.812	$[M+H]^+$ 353.0876	Polyphenols
21	Catechin	$C_{15}H_{14}O_6$	290.07892	4.973	$[M-H]^-$ 289.07153	Polyphenols
22	Corilagin	$C_{27}H_{22}O_{18}$	634.0808	5.101	$[M-H]^-$ 633.07373	Polyphenols
23	Procyanidin B2	$C_{30}H_{26}O_{12}$	578.16456	5.139	$[M-H]^-$ 577.15729	Polyphenols
24	(+)-Catechin hydrate	$C_{15}H_{14}O_6$	290.07904	5.234	$[M-H]^-$ 289.07178	Polyphenols
25	p-Coumaric acid	$C_9H_8O_3$	164.04739	5.27	$[M-COOH]^-$ 119.04921	Phenolic acids
26	Caffeic acid	$C_9H_8O_4$	180.04217	5.364	$[M-H]^-$ 179.03500	Organic acid
27	(1R,3R,4S,5R)-1,3,4-trihydroxy-5-[(E)-3-(4-hydroxyphenyl)prop-2-enoyl]oxycyclohexane-1-carboxylic acid	$C_{16}H_{18}O_8$	338.10020	5.414	$[M-H]^-$ 337.0929	Phenolic acids
28	Quinic acid	$C_7H_{12}O_6$	192.06334	5.42	$[M-H]^-$ 191.05606	Polyphenols

29	Emodin	$C_{15}H_{10}O_5$	229.02609	5.501	$[M+CAN+H]^+$ 271.05991	Anthraquinones
30	Procyanidin B1	$C_{30}H_{26}O_{12}$	578.14208	5.683	$[M+H]^+$ 579.1495	Polyphenols
31	Rutin	$C_{27}H_{30}O_{16}$	610.1535	5.762	$[M-H]^-$ 609.14661	Flavonoids
32	Naringenin-7-O-glucoside	$C_{21}H_{22}O_{10}$	434.12144	5.829	$[M-H]^-$ 433.1136	Flavonoids
33	Naringenin	$C_{15}H_{12}O_5$	272.06844	5.895	$[M+H]^+$ 273.07574	Flavonoids
34	Quercetin 7-rhamnoside	$C_{21}H_{20}O_{11}$	448.10047	5.926	$[M-H]^-$ 447.05670	Glycosides
35	Quercetin-3β-D-glucoside	$C_{21}H_{20}O_{12}$	464.09546	5.936	$[2M-H]^-$ 927.18268	Glycosides
36	Kaempferol-7-O-beita-D-glucopyranoside	$C_{21}H_{20}O_{11}$	448.10057	6.055	$[M-H]^-$ 447.056895	Glycosides
37	Quercetin-3-O-xyloside	$C_{20}H_{18}O_{11}$	434.0848	6.15	$[M-H]^-$ 433.07645	Glycosides
38	Pinoresinol 4-O-glucoside	$C_{26}H_{32}O_{11}$	520.19472	6.153	$[M-H+HAc]^-$ 579.20825	Glycosides
39	Cynaroside	$C_{21}H_{20}O_{11}$	448.10051	6.168	$[M+H]^+$ 449.10767	Flavonoids
40	Ferulic acid	$C_{10}H_{10}O_4$	194.05789	6.28	$[M-H]^-$ 193.05067	Organic acid
41	taxifolin	$C_{15}H_{12}O_7$	304.05827	6.308	$[M-H]^-$ 303.05081	Flavonoids
42	Astilbin	$C_{21}H_{22}O_{11}$	450.11629	6.356	$[M-H]^-$ 449.10861	Flavonoids
43	Eriodictyol	$C_{15}H_{12}O_6$	288.06332	6.363	$[M+H]^+$ 289.07037	Polyphenols
44	Ligustilide	$C_{12}H_{14}O_2$	190.09931	6.397	$[M+H]^+$ 191.10652	Phthalide compounds

45	Phloretin-2'-O-glucoside	$C_{21}H_{24}O_{10}$	436.13699	6.435	$[M-H]^-$ 435.1297	Glycosides
46	Quillaic acid	$C_{30}H_{46}O_5$	486.33442	6.567	$[M-H]^-$ 487.34152	Terpenoids
47	Linderalactone	$C_{15}H_{16}O_3$	203.08349	6.582	$[M+CAN+H]^+$ 245.11734	Sesquiterpene Lactones
48	Caffeyl alcohol	$C_9H_{10}O_3$	166.06302	6.626	$[M-H]^-$ 165.05574	Terpenoids
49	3-phenyllactic acid	$C_9H_{10}O_3$	120.05744	6.637	$[m+FA-H]^-$ 165.05557	Organic acid
50	18 β-Glycyrrhetintic Acid	$C_{30}H_{46}O_4$	470.33927	6.667	$[M+H]^+$ 471.34613	Terpenoids
51	Tiliroside	$C_{30}H_{26}O_{13}$	297.06863	6.787	$[2M+H]^+$ 14453	Glycoside flavonoids
52	Dihydrokaempferol	$C_{15}H_{12}O_6$	288.06344	6.843	$[M-H]^-$ 287.0660	Flavonoids
53	Camphor	$C_{10}H_{16}O$	152.12005	6.956	$[M+H]^+$ 153.12729	Terpenoids
54	Pedunculoside	$C_{36}H_{58}O_{10}$	696.40883	7.138	$[M-H]^-$ 659.40137	Saponins
55	Rosamultin	$C_{36}H_{58}O_{10}$	650.40333	7.166	$[M+FA-H]^-$ 668.4358	Terpenoids
56	Quercetin	$C_{15}H_{10}O_7$	302.0425	7.174	$[M-H]^-$ 301.0353	Flavonoids
57	Eupafolin	$C_{16}H_{12}O_7$	316.05825	7.184	$[M-H]^-$ 315.0511	Flavonoids
58	Medicagenic acid	$C_{30}H_{46}O_6$	502.32936	7.296	$[M+H]^+$ 503.33667	Terpenoids
59	Ethyl caffeate	$C_{11}H_{12}O_4$	208.07349	7.305	$[M-H]^-$ 207.06631	Polyphenols
60	Ursolic acid	$C_{30}H_{48}O_3$	456.36042	7.349	$[M- COOH]^-$ 411.36230	Terpenoids
61	Naringenin chalcone	$C_{15}H_{12}O_5$	272.06847	7.423	$[M-H]^-$ 271.0611	Flavonoids

62	Medicagenic acid	$C_{30}H_{46}O_6$	502.32932	7.423	$[M-COOH]^-$ 457.33136	Terpenoids
63	Ilexsaponin A	$C_{36}H_{56}O_{11}$	664.38209	7.435	$[M+NH4]^+$ 682.41583	Saponins
64	Ursonic acid	$C_{30}H_{46}O_3$	454.34475	7.596	$[M-COOH]^-$ 409.34641	Terpenoids
65	5,6,7-trihydroxy-2-(4-methoxyphenyl)-4H-chromen-4-one（KAEMPFERIDE）	$C_{16}H_{12}O_6$	300.06333	7.623	$[M+H]^+$ 301.07059	Flavonoids
66	Oleanonic acid	$C_{30}H_{46}O_3$	454.34462	8.114	$[M+H]^+$ 455.35153	Terpenoids
67	Pinocembrin	$C_{15}H_{12}O_4$	256.07346	8.41	$[M-H]^-$ 255.08613	Flavonoids
68	Wogonin	$C_{16}H_{12}O_5$	284.06838	8.666	$[M-H]^-$ 283.06107	Flavonoids
69	Lindenenol	$C_{15}H_{18}O_2$	230.13079	9.417	$[M+H]^+$ 231.13805	Terpenoids
70	Maslinic acid	$C_{30}H_{48}O_4$	472.3553	10.141	$[M+H]^+$ 473.36243	Terpenoids
71	Linolenic acid ethyl ester	$C_{20}H_{34}O_2$	306.25569	10.271	$[M+H]^+$ 306.25569	Aliphatic acid
72	Oleanolic acid	$C_{30}H_{48}O_3$	438.35	11.833	$[M+H]^+$ 439.3572	Terpenoids

Supplementary Table S2 Sequences of primers used in this study.

Gene	Primer sequwnces
GAPDH	F: 5'-ATGGTGAAGGTCGGTGTGAA-3'<br>R: 5'-CCTTGACTGTGCCGTTGAAT-3'
Cxcl 3	F: 5'-TGAGGCAGTATTCCTTGGCTG-3'<br>R: 5'-ACCGGCATGACCTTGTTTGT-3'
IL-17a	F: 5'-TGGACTCTCCACCGCAATGAAG-3'<br>R: 5'-GCTTTCCCTCCGCATTGACAC-3'
MMP 13	F: 5'-TTGTGTTTGCAGAGCACTACTTGA-3'<br>R: 5'-AACTGTGGAGGTCACTGTAGACTTCTT-3'
MMP 3	F: 5'-GACGATGATGAACGATGGACAGAGG-3'<br>R: 5'-TGTGGAGGACTTGTAGACTGGGTAC-3'
Traf 6	F: 5'-TACGATGTGGAGTTTGACCCA-3'<br>R: 5'-CACTGCTTCCCGTAAAGCCAT-3'
S100a8	F: 5'-GACAATGCAATTAACTTCGAGGAG-3'<br>R: 5'-TGTGGCTGTCTTTGTGAGATGC-3'
SOD 1	F: 5'-TAACTGAAGGCCAGCATGGGT-3'<br>R: 5'-GGTCTCCAACATGCCTCTCTTC-3'
SOD 2	F: 5'-CAGACCTGCCTTACGACTATGG-3'<br>R: 5'-GCTGAAGAGCGACCTGAGTTGT -3'
GPX 2	F: 5'-GAACGAGGAGATCCTGAACAGC-3'<br>R: 5'-GGTAGGGCAGCTTGTCTTTCAG-3'
PPARγ	F: 5'-CAGGCTTGCTGAACGTGAAG-3'<br>R: 5'-GGAGCACCTTGGCGAACA -3'

S100a9	F: 5'-GGAAGCACAGTTGGCAACCTTTATG-3'<br>R: 5'-ACCGGCATGACCTTGTTTGT-3'
Lcn2	F: 5'-GGCTGTCGCTACTGGATCAGAAC-3'<br>F: 5'-CGAACTGGTTGTAGTCCGTGGTG-3'
Hsd17b13	F: 5'-CAGACCTGCCTTACGACTATGG-3'<br>R: 5'-AACTGTGGAGGTCACTGTAGACTTCTT-3'
Akt4	F: 5'-CATCACTGGTGAGCCACTTGTCC-3'<br>R: 5'-CCGCATCCTTGTACCGTCTTAGC-3'
Cyp2d26	F: 5'-CAGACCTGCCTTACGACTATGG-3'<br>R: 5'-AACTGTGGAGGTCACTGTAGACTTCTT-3'
MUC1	F: 5'-AGTGCCAAGTCAATACCCTGT-3'<br>R: 5'-CTGGGGTGAACTGTTACTGGA-3'
COX 2	F: 5'- CTGGTGCCTGGTCTGATGATGTATG-3'<br>R: 5'-GGATGCTCCTGCTTGAGTATGTCG-3'
Occludin	F: 5'-TGGCTATGGAGGCGGCTATGG-3'<br>R: 5'-AACTGTGGAGGTCACTGTAGACTTCTT-3'
IL-6	F: 5'-CTTCTTGGGACTGATGCTGGTGAC-3'<br>R: 5'-TCTGTTGGGAGTGGTATCCTCTGTG-3'
TNF-α	F: 5'-CGCTCTTCTGTCTACTGAACTTCGG-3'<br>R: 5'-GTGGTTTGTGAGTGTGAGGGTCTG-3'
IL-1β	F: 5'-ATGGTGAAGGTCGGTGTGAA-3'<br>R: 5'-TGTCGTTGCTTGGTTCTCCTTGTAC-3'

Supplementary Table S3 Offline data statistics

Sample	Reads NO.	Bases (bp)	Q30 (bp)	Q30 (%)
HB78	41006920	6192044920	5787181331	93.46
HB88	39645274	5986436374	5601663487	93.57
HB5	46606664	7037606264	6579323406	93.48
MB32	40269834	6080744934	5708059053	93.87
MB90	38849132	5866218932	5454412417	92.98
MB38	40423358	6103927058	5717318717	93.66
CB15	42719252	6450607052	5983472586	92.75
CB18	40256634	6078751734	5684456045	93.51
CB31	45395988	6854794188	6368425260	92.90

Reads NO. : Total Reads; Bases (bp): Total number of bases; Q30 (bp): The total number of bases with a base recognition accuracy of over 99.9%; Q30 (%): The percentage of bases with a base recognition accuracy of 99.9% or higher.
